# Analysis and Predictability of Drought In Northwest Africa Using Optical and Microwave Satellite Remote Sensing Products

**DOI:** 10.1038/s41598-018-37911-x

**Published:** 2019-02-06

**Authors:** Michel Le Page, Mehrez Zribi

**Affiliations:** CESBIO, Université de Toulouse, IRD/CNRS/CNES/INRA/UPS, 8 Av. Edouard Belin, 31401 Toulouse cedex 9, France

## Abstract

In a context of high stress on water resources and agricultural production at the global level, together with climate change marked by an increase in the frequency of these events, drought is considered to be a strong threat both socially and economically. The Mediterranean region is a hot spot of climate change; it is also characterized by a scarcity of water resources that places intense pressure on agricultural productivity. This article analyzes the potential for using multiple remote sensing tools in the quantification and predictability of drought in Northwest Africa. Three satellite products are considered: the Normalized Difference Vegetation Index (NDVI), Soil Moisture Index (SWI), and Land Surface Temperature (LST). A discussion of the variability of these products and their inter-correlation is presented, illustrating a generally high consistency between them. Statistical anomaly indices are then computed and a drought severity mapping is presented. The results illustrate in particular a high percentage of dry conditions in the region studied during the last ten years (2007–2017). Finally, we propose the use of the analog statistical approach to identify similar evolutions of the three variables in the past. Although this technique is not a forecast, it provides a strong indication of the plausible future trajectory of a given hydrological season.

## Introduction

The quantification, monitoring and identification of the beginning and end of droughts as well as their extension is a highly complex task^1^. In recent decades, various indices have been developed to answer these questions. These indices are usually based on precipitation and other meteorological variables^2–9^. Despite the potential of the proposed indices, the distribution and density of the measuring stations are generally insufficient to allow a spatialization of the proposed drought indices. This is particularly the case in less developed countries with generally limited ground instrumentation and also limited statistics on precipitation measurements.

In recent years a number of scientific studies have developed indices based on time series of remote sensing satellite products^10–19^. These spatial measurements using various spatial techniques (optical and microwave measurements) are directly related to surface features such as vegetation cover, soil moisture or surface temperature.

For vegetation cover, measurements are mostly based on optical remote sensing. More specifically, the NDVI satellite index, often used in vegetation analysis, is strongly correlated with vegetation cover growth^20^. Using NDVI from several types of optical sensors (AVHRR, SPOT-VGT, MODIS etc.), various normalizations along the time axis have been proposed to study the anomalies, and in particular drought. The most popular is VCI, ranging between 0 to 1 (0 for the driest condition, 1 for the wettest) applied in several regions of the globe to estimate agricultural drought under varying ecological conditions^10,11^. It can give the status of vegetation in comparison to the best and worst vegetation conditions for a particular monthly period over many years. Amri *et al*.^18^ have proposed the VAI parameter, which provides a comparison of the mean value for a particular period normalized against the standard deviation of data acquired over many years. It has been successfully tested over a number of sites (North Africa, etc.).

For soil water, various active or passive microwave techniques have offered estimates of the soil surface water content^21,22^. Based on empirical or physical approaches, the surface estimation has enabled estimation of the water content in a soil profile^20^. In recent years, with the advent of many time series of satellite products increasingly offering these land cover estimates or information on soil water content (ASCAT, SMOS etc.), a number of studies have proposed new drought indices based mainly on analyses of statistical anomalies over several years. Amri *et al*.^15^ proposed the MAI index based on anomaly analysis using the ASCAT SWI moisture product. It showed a strong correlation with the SPI index over the central region of Tunisia. Sanchez *et al*.^23^ proposed an agricultural drought index SMADI based on SMOS data coupled to a MODIS product, applied over Spain.

In the same context, other composite indices based on the analysis of several types of index have been proposed. For example, Mu *et al*.^19^ proposed the Drought Severity Index DSI, derived from evapotranspiration and NDVI anomalies. After recalling the drawbacks of the PDSI index^24^ and particularly the uncertainty of precipitations they also note that the DSI is subject to uncertainties including the global reanalysis input data and the MODIS Evapotranspiration algorithm.

In addition to this estimation of drought intensity using drought indices, it remains essential for stakeholders to predict the future dynamic of a drought event in relation to specific drought conditions. Various studies have been proposed to address this question, particularly by analyzing precipitation levels. The analogue technique for has been widely used in climatology. After the Second World War, Howe^25^ used the technique to look for agro-climatic analogues with the objective of introducing new plants from a region to another based on their similar climates. Howe described climatic analogues as “*areas that are enough alike with respect to some of the major weather characteristics affecting crop production, particularly during the growing period, to offer a fair chance for the success of plant material transplanted from one area to its climatic counterpart*”. More recently, some studies have used the analogue technique to look for analogous regions in the context of climate change. Hallegate *et al*.^26^ searched for the location of analogous future climates for European cities. Pugh *et al*.^27^ also looked for the locations of analogous climates, but used the results to analyze the potential impact on croplands. CGIAR^28^ has proposed a tool based on the same approach.

Meteorologists have also explored this technique for short-term forecasting. In early studies, Lorenz^29^ and Ruosteenoja^30^ found that the likelihood of finding perfect analogues is low, so that an extremely long historical time series would be needed. They concluded that the performance of this technique for meteorological forecasting is very poor compared to numerical models. Nevertheless, Van Den Dool^31^ found that there is some potential in Analog Forecasting, and more recently Hamill and Whitaker^32^ found reasons to believe that the Analog Forecast is competitive with high-resolution forecast models.

Barnett and Preisendorfer^33^ proposed a theoretical framework in which a climate state vector represents the evolution over time of the climate system and examined three techniques for selecting analogues. Hamill and Whitaker^32^ proposed a general theory for the statistical correction of weather forecasts based on observed analogues. These authors described ten different techniques of looking for analogues. The basic idea is to compute a distance between a reference vector and other state vectors. The variables composing the vector can also be assigned different weights. The search region can be limited spatially and temporally. For example, an analogue can be sought within a distance of less than 100 km, and within a period of less than 45 days. The distance metric is generally a Euclidean distance, but other metrics such as Mahalanobis can also be used.

The objective of the paper is to analyze the potential for using multiple remote sensing tools in the quantification and predictability of drought in Northwest Africa. After presenting the study area, we study the Correlation and auto-correlation of drought indicators. We then map the drought intensity and analyze the predictability of drought using the analogue search technique. The data and methods are explained in a last section coming after the conclusion.

## Area of Study

**Figure 1:**
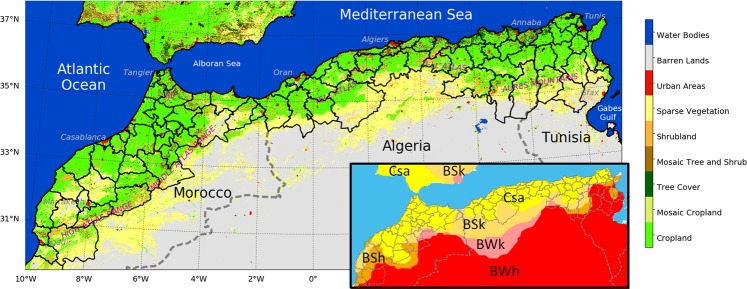
The study area with its 90 administrative areas and a simplified land cover classification extracted from the ESA-CCI Land cover of 2010. The inlay map shows the Koeppen-Geiger classification of climate for the area.

The area studied is located in the productive region of Northwest Africa in the countries of Morocco, Algeria and Tunisia, also known as the Maghreb. Aside from its cultural roots, this region also shares a common geography, with a narrow coastal plain separated from the Sahara by mountain ranges: the High and Middle Atlas in Morocco, the Tell Atlas in Algeria, and the Aures mountains on the east of the region (Fig. 1). According to the Köppen-Geiger classification^34^, the climate of the study area is mostly Mediterranean (Csa) and Semi-Arid (BSk and BSh). Most of its 90 million inhabitants and the productive agricultural areas are located on the coastal plains. Figure 1 is a simplified map of land cover based on the ESA CCI 2010 product (https://www.esa-landcover-cci.org) that highlights the cropland area. The climate of the region is influenced by several climatological regimes: the Mediterranean regime, the North Atlantic regime and finally that of the Sahara in the south^35^. Thus, this region is characterized by a climate with strong spatial and temporal variability. The Mediterranean region and particularly North Africa is considered to be a hot spot of climate change^36^. It is regarded as a particularly vulnerable zone owing to its hydraulic poverty^37^ linked to the rarity and the high temporal variability of precipitation. These climatic conditions and particularly the expected increase in drought events could have a highly detrimental effect on agricultural productivity and on biodiversity. In this region, drought events may affect the recharge of reservoirs and groundwater, essential for irrigation and domestic water use.

The typical agricultural landscape of this Mediterranean area is composed of cereals and olive trees. Cereals (wheat, barley and oats) are the most widely produced crop in the area and cover approximately 46% percent of the cultivated area. Olive orchards represent approximately 14% of the agricultural area, but are very dominant in Tunisia, with 2 million hectares. Other orchards (citrus, grapes) and vegetables (potatoes, chili, watermelon…) are also common.

**Figure 2:**
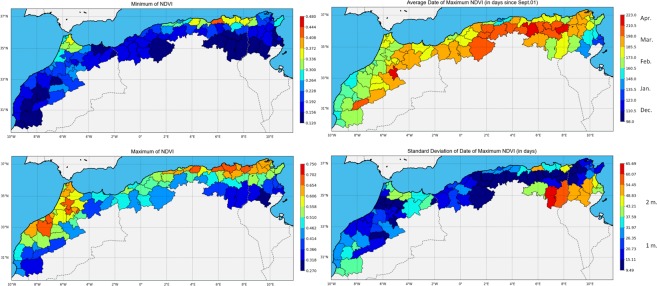
NDVI analysis. Top left: Maximum NDVI, bottom left: Minimum NDVI, Top right: Date of Maximum NDVI, Bottom right: Standard deviation of the date of Maximum NDVI.

The two maps on the left of Fig. 2 show the minimum and maximum NDVI (MODIS MOD13Q1 product) for the period 2001–2018, presented as a mean value for each administrative area. The region is not homogeneous; in particular, the minimum NDVI map shows that the region of Tangier (northern Morocco) and the northeast of Algeria never fall below 0.3, typical of perennial forests and higher annual rainfalls. The maximum NDVI map also reveals two intensive cropland areas in western Morocco around the Settat and Meknes regions and in northern Tunisia. The north-to-south and west-to-east gradients in Morocco are also noticeable on the maximum NDVI map. In the south of the study area both minimum and maximum NDVI reach lower values because of increasingly semi-arid to arid conditions.

The two maps on the right of Fig. 2 show the date of maximum NDVI and its standard deviation. On the west side of the study area, there is a clear gradient between early maximums in late January on the Atlantic coast to the end of March in the inland areas. In Algeria the gradient is west to east and north to south with NDVI apogee ranging from the end of February to the beginning of April. Finally the gradient is inverted on the coast of Tunisia where the maximum NDVI can occur as early as the end of December. The standard deviation map shows that the date of maximum NDVI has little variation overall (<1 month), but may reach as much as two months in the more arid regions of Tunisia. These discrepancies can be explained by two factors. First, major rainfall in autumn can generate pasture growth from November onwards. Second, the agricultural cycle generally attains maximum growth between March and April for dominant crops such as cereals.

## Results

### Correlation and auto-correlation of drought indicators

**Figure 3:**
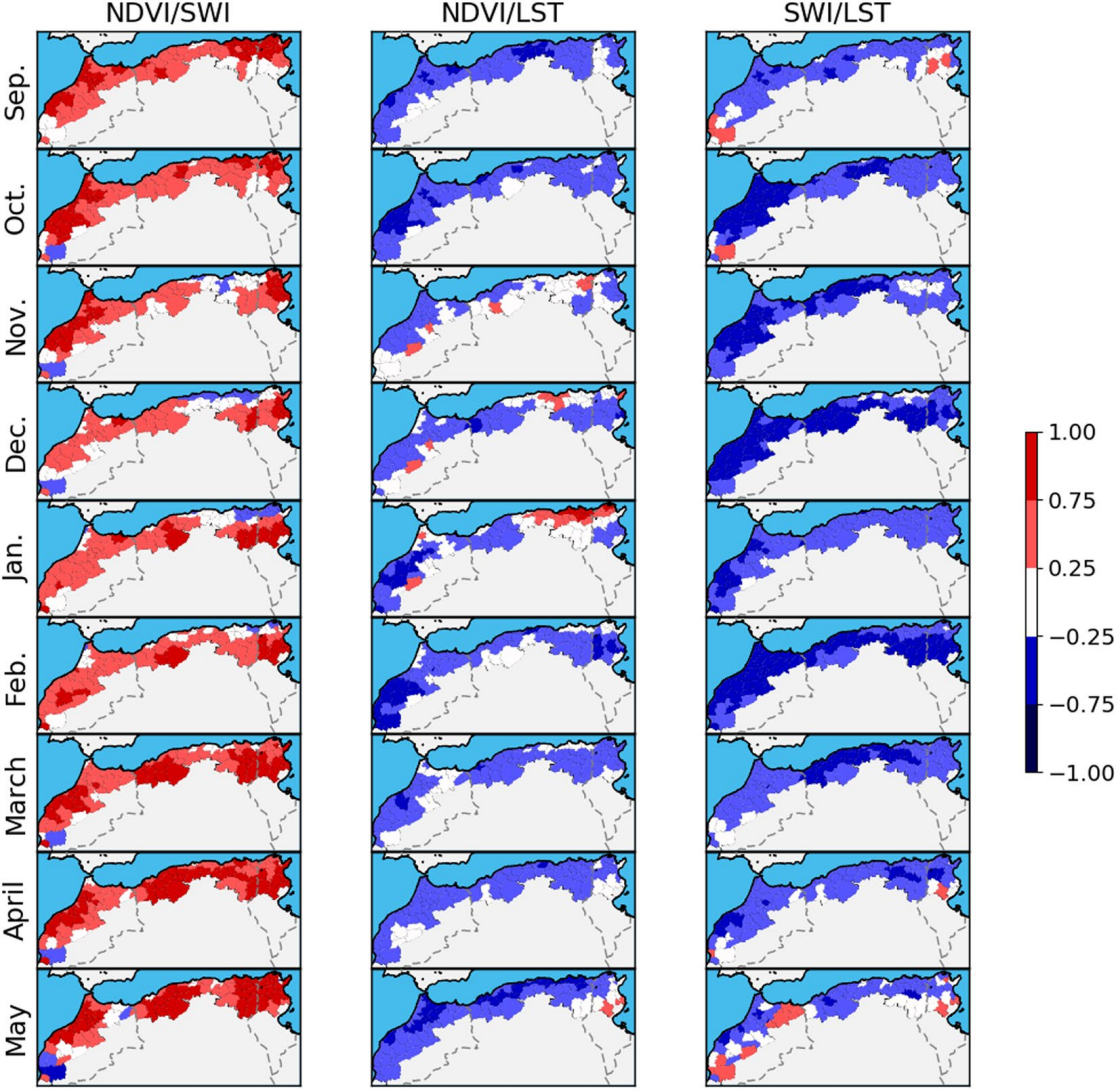
Correlation between the three drought indices from September to May between 2007 and 2018.

Figure 3 shows the correlation between the three couples of variables (NDVI/SWI, NDVI/LST and SWI/LST) between September and May, using the 11 years of data. The correlation between NDVI and SWI is generally high, but weakens during the winter months. On average for the whole area, the weaker correlations are in December and January (0.27 and 0.33) while the average correlations are above 0.57 for the months of September, October, and March to May. Furthermore, the correlations are near null or even significantly negative in the more wooded areas of northeast-Algeria and north Tunisia. The limited correlations during December and January could be explained by the limited precision of SWI satellite products in forest areas where the effect of vegetation cover on microwave signals is dominant. It can also be explained by important changes linked to precipitations during this period. The moisture level increases first before the vegetation growth (pastures, winter agricultural covers). In several areas in the south, we also observe a weaker correlation that could be explained simply by the limits of statistical analysis for areas with very sparse vegetation cover without agriculture.

The negative correlation between NDVI and LST is explained by the reduction of surface temperature due to the higher evapotranspiration of the vegetation cover in agricultural areas. However, the behavior of correlation is similar to the previous one with a decrease of correlation in the winter month. A positive correlation is observed in the north east of the study area that could be explained by the forest leaf loss simultaneously to the decreasing of surface temperature (the coldest months).

Logically, SWI and LST are also anticorrelated. No coherent behaviors are observed during the driest months (September, May) due to the extreme low moisture values and high changes in temperature (hottest months).

**Figure 4:**
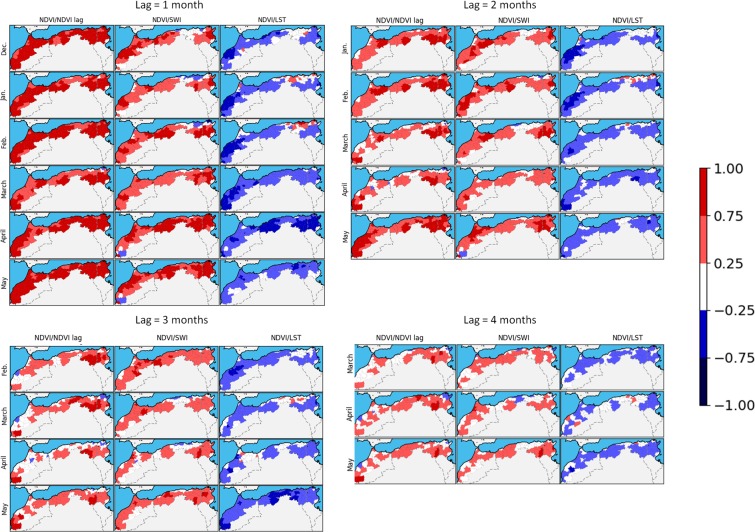
Lagged correlations between NDVI and lagged NDVI, and lagged SWI and lagged LST.

Zribi *et al*.^38^ have illustrated a strong relationship between the soil moisture SWI variable and the NDVI retrieved one month later. In this context, Fig. 4 illustrates the correlation between SWI and NDVI retrieved with a lag of one to four months. As expected, correlations decrease with longer lag, but the auto-correlation can still be high after four months. Based on this analysis, the behaviors of the different variables and their correlation, it seems sufficient to employ the two variables NDVI and SWI for future studies concerning drought mapping.

### Mapping of drought intensity

**Figure 5:**
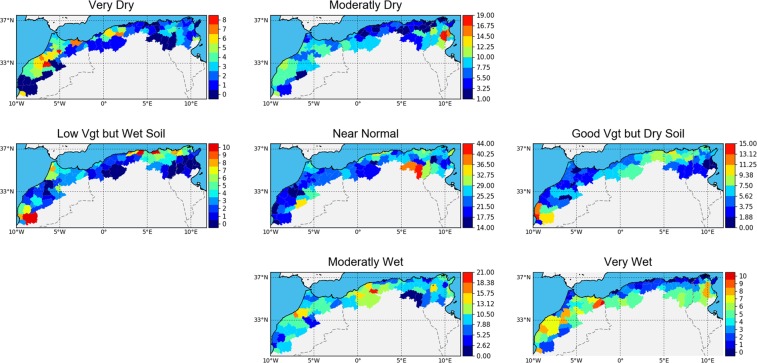
Drought Classification. Each map shows the number of occurrence of each situation between November and April.

Based on the defined Drought vector with VAI and MAI as drought index elements, the results of the threshold classification discussed in the methodology section are presented in Fig. 5, which shows the number of occurrences of a situation for the months November through May from 2007 to 2018. Each occurrence map ranges from dark blue (lower occurrence) to red (higher occurrence). Overall, the “Near Normal” situation (central map) is the most common situation (51.3%), nevertheless the red colors show areas where this situation occurred more often (east of Algeria for example), whereas it happened more rarely in dark blue areas such as Morocco for example. The moderately dry situation occurred throughout the studied areas, but with more occurrences in the semi-arid regions of Morocco and Tunisia. Very Dry situations occurred mainly in Morocco and central Algeria, but are less frequent.

**Figure 6:**
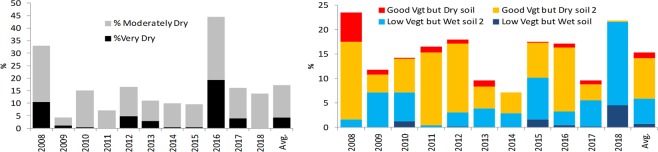
Left: Percentage of Dry classes; Right: Percentage of ambiguous classes, for the months of December through April.

The composition of the classes can be analyzed for their monthly and yearly distributions. The monthly distribution (not shown) shows little variation of the classes over the individual months. Figure 6-Left shows the percentages of the classes ‘Very Dry’ and ‘Moderately Dry’ for each year (which consists of 5 months for each of the 90 areas). The figure suggests that the years 2007–08 and 2015–16 were particularly dry with respectively 1.7% very dry and 25.4% moderately dry and 8.8% very dry, and 32.7% moderately dry.

Ambiguous situations are the less common classes (13.2%), but provide general information about a trajectory change during the growing season. Figure 6-Right shows the percentages of the four contradictory classes. Most of the years display a more or less equal number of moderately ambiguous situations. Some years have a specific behavior. The year 2008 has a large proportion of “Good vegetation but dry soils”, both in the extreme and moderate classes. This suggests that this year began with a propitious situation for crops, but later reverted to a dry situation. The proportions indicate the opposite for the year 2018. There is a majority of low levels of development of the vegetation with wet soils, which suggests that a dry situation reverted to a favorable one. An examination of the monthly distribution of ambiguous situations (not shown), suggests that such inversions are more common during the months of January and February: the average monthly occurrence of those situations is 12%, whereas it is more than 17% in January and over 20% in February, which suggests that those two months are decisive for the evolution of a drought situation in this region. Such observed ambiguous situations could be a helpful indicator for identifying the beginning or end of a drought period, often very difficult to determine.

### Predictability analysis using the analogue test

In a context of drought conditions, as discussed in the introduction, the stress on agriculture and productivity becomes very high. Stakeholders and deciders need tools for evaluating the drought’s intensity, but also an evaluation of the possible future trajectory of the agricultural season. We propose a situation analysis based on an algorithm of resemblance that uses statistics to identify previous seasons whose behavior closely compares to the current one.

The results of these analogue tests over the entire area are summarized in Table 1, which shows the tests’ performance in finding analogous years for the drought vector VD over the months November through April. In accordance with the auto-correlation study, the closer we are to the tested month (April), the better the score. Moreover, even if extended to 6 months earlier (November), the prediction is still statistically significant. In fact, this test shows that the scores for November, while low, are still about twice as good as those for a random year. By January the score reaches almost 50% for the first analog and 72% between the first and second analog, meaning that by January we have almost a 75% chance of having identified the most analogous year. By February, there is an 80% chance that the most similar year falls between the two selected ones. Table 1 also shows the average rank of the analogue years, which is a good indicator of how many years to take into account when looking for resembling years.

**Table 1:** Performance of analogue retrieval.

	Percentage of First Analog	Percentage of First or Second Analog	Average Rank
November	21.2	38.4	4.09
December	32.6	53.0	3.12
January	47.5	72.4	2.46
February	57.3	80.0	2.03
March	71.4	91.7	1.51
April	100	100	1

The lower performances in November and December are easily explained by the great uncertainty of rainfall during those months that could trigger the development of vegetation. The average percentage of first analogue in January and February can still be attributed to the uncertainty of rainfall but also to ambiguous situations, as seen earlier. Moreover the very small number of historic situations that we can examine is obviously a drawback for the method. There is a high probability of falling upon unknown trajectories. In such cases, the ranked analogues are not similar to the studied situation, but a threshold above the computed distance would allow us to eliminate those cases.

### Case studies of trajectories

The analogue selection is not a prediction in itself; in fact the various ranked years may have very different trajectories. However, examination of the proposed years can provide valuable information for an analyst. If the selected years resemble each other, the analyst visually infers an average trajectory. But if the proposed years are dissimilar, the analyst visually observes the uncertainty of the prediction.

In this section, we analyze the results of the classification scheme and the analogue test on selected areas for the specific years of 2007–08, 2015–16 and 2017–18. We selected three contrasting administrative areas from our study region. The climate of the Moroccan province of Settat is mostly influenced by the Atlantic Ocean, the Wilaya of Aïn Defla is a mountainous area in the Tell range that is the leading Algerian producer of potatoes, and the governorate of Kairouan in Tunisia, which is 50 km from the Mediterranean, has a more semi-arid climate.

**Figure 7:**
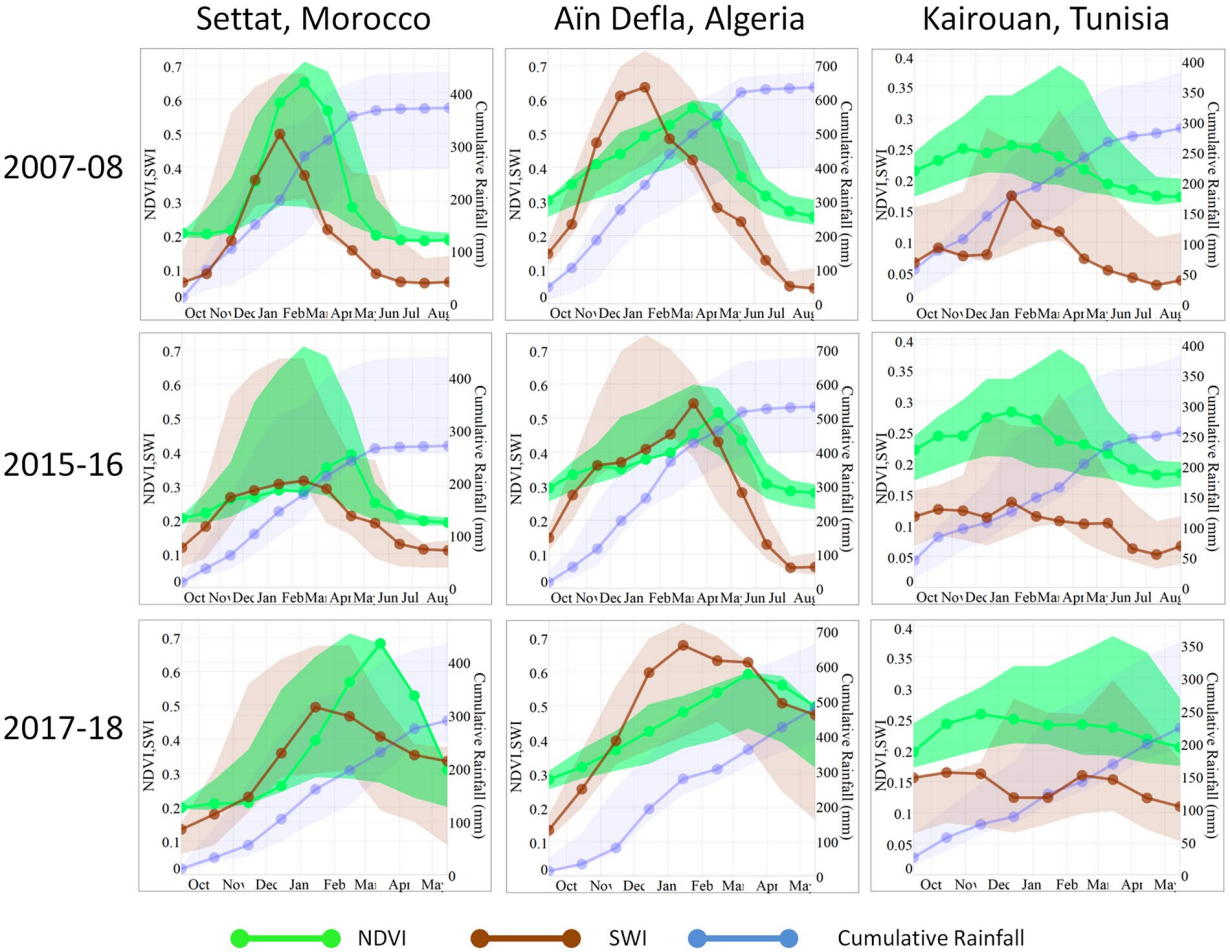
Trajectories of NDVI, SWI and cumulative rainfalls for the administrative areas of Settat (Mor.), Aïn Defla (Alg.), Kairouan (Tun.).

Figure 7 illustrates the trajectories of NDVI and SWI over a year, clearly showing the spatial and temporal heterogeneity of the study area. The cumulative rainfalls extracted from the CHIRPS (Climate Hazards Group InfraRed Precipitation with Station data) product are also plotted. Additionally, the maximum and minimum values of each month for the whole time series of each variable (2000–2018 for NDVI) (2007–2018 for SWI and 1981–2018 for rainfalls) is plotted so that the reader can easily infer the anomalies. The trajectories are very different for each region, even for the same year.

We will consider the year 2017–18, which was previously characterized by a large content of the classes “Low vegetation but dry soils” and a small percentage of “Moderately Dry”. On the trajectories, we can see several behaviors. In Settat the season begins with low soil moisture, so that the vegetation remains at a low level; then in January/February the soil moisture increases dramatically so that the development of vegetation finally reaches a maximum in March, one month later than the average maximum date. The season’s duration of vegetation is reduced, owing to this late start. In Aïn Defla, the season does not include any kind of drought. In Kairouan, the development of vegetation is weak, but not very different from the two other years.

**Figure 8:**
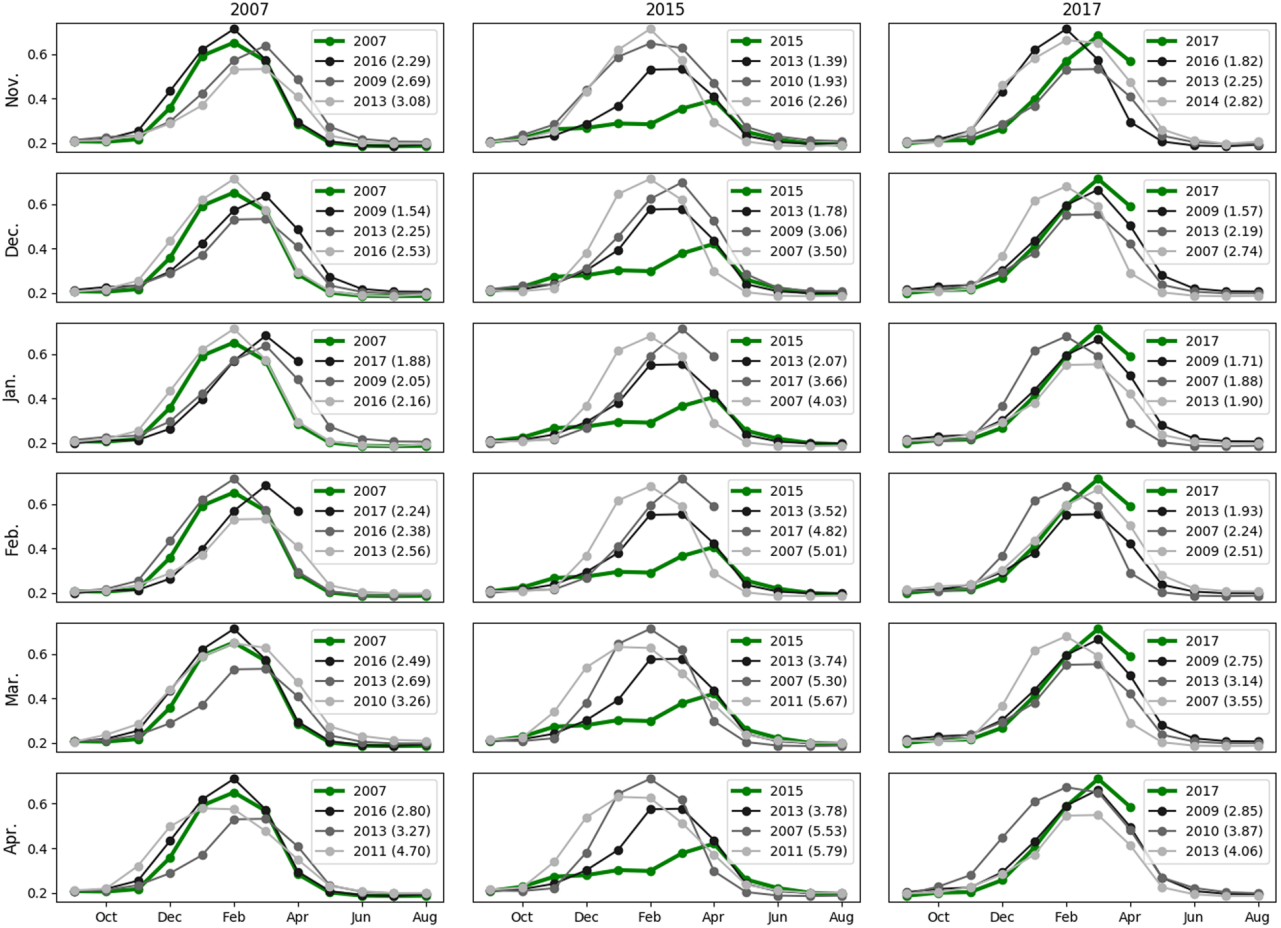
Analogue tests for Settat, Morocco.

Figure 8 illustrates the results of the analogue test for the area of Settat, Morocco. The plot is presented as a grid where the columns correspond to the three specific years of 2007–08, 2015–16 and 2017–18, and the rows correspond to the tested months from November through April. Each plot shows the trajectories over a full hydrological year (Sept-Aug) of NDVI, and the NDVI trajectories of the first three analogues obtained for the tested month. The Euclidean distance is shown between parentheses in the legend. The two other variables of the drought vector that were used for the computation are not shown. For the year 2007–2008, the best analogue is the year 2016. In the previous months, the year 2016 consistently appears to be one of the three most typical years. In November, the first analogue is also 2016, but as can be seen on this graph the three preceding months are very similar, so that the scores are also very close. In December 2007 and January 2008, the algorithm chose the year 2009 as a possibility, but with a longer duration this possibility disappears. Overall, the two more similar years of 2016 and 2013 were selected 6 and 5 times out of 6.

The year 2015–16 was very dry. The most remarkable information from the analog time series is that there is no other similar year in the 2007–2018 dataset. This is shown by the values of the Euclidean distance: In November and October, the score remains low (1.39 and 1.78), but it then increases progressively until the month of April. Despite those bad scores, the most similar year was always the year 2013, suggesting that 2015–16 would be at least as dry as 2013.

The year 2017–18 shows all the signs of the onset of a drought but with a late season of rain, which canceled the drought. The closest year, 2009, was detected as early as December. Yet in February the ranking was shuffled suggesting that there could still be a change of trajectory.

## Conclusion

This paper proposes an analysis of drought phenomena in Northwest Africa using multi-sensor time series satellite products. In fact, despite certain limitations linked to measurement length and frequency, the satellite indices could be a useful tool, complementary to the traditional indices particularly based on precipitation statistics, for the analysis of drought situations. This is especially the case in areas where the rain gauge network is sparse.

Three variables are considered, the NDVI, the SWI and the LST. A very strong annual correlation is observed between NDVI and SWI for almost all the areas considered in the region of study. This correlation decreases for forest areas, for which satellite measurements to estimate soil moisture are not sufficiently precise, and in southern areas with very dispersed vegetation cover and limited agriculture. Monthly analysis illustrates more or less the same behavior, with the highest correlations generally found at the time of maximum growth during the agricultural season, approximately around March and April. The correlation between LST and the two other variables is also discussed. In this case we observe a correlation close to −1, particularly for agricultural areas. Based on the results of correlation a mapping of drought conditions is proposed, using just the two drought indices VAI and MAI. Seven classes of drought are considered (very wet, wet, normal, dry, very dry, wet soil and low vegetation, and dry soil and good vegetation). The results of classification using the drought vector show the presence of moderate drought and very dry conditions in certain parts of the studied site for all the years examined. The longest periods of drought are found in the driest years: 2008 and 2016. The ambiguous situations corresponding to wet soil and low vegetation or dry soil and high vegetation are particularly seen in the two months of January and February. These specific conditions could be helpful factors in identifying the beginning or the end of a drought period. A predictability analogue algorithm is proposed in order to identify statistically the year in the past closest to the conditions of a present year, based on the drought vector with VAI and MAI indices and a database of eleven years (2007–2018). The results illustrate the high potential of this approach for retrieving analogous years. The capacity for retrieving an analogous year processed from December (beginning of the agricultural season) through April increases with time. In February for example, the percentage of the first or second analogue reaches 80%. This type of result could be very helpful to stakeholders to better situate the drought conditions of one year as compared to past years with known returns on agricultural production and water reserves.

A discussion of three studied areas (Settat in Morocco, Ain Defla in Algeria and Kairouan in Tunisia) illustrates the behaviors of the VAI and MAI indices and their correlation. Particular attention was given to the analysis of ambiguous situations that turned out to be reversals.

The analysis proposed in this study over the whole of Northwest Africa could be implemented operationally on a platform called MEDI (http://osr-cesbio.ups-tlse.fr/medi) well suited for use by water managers and stakeholders.

## Data and Methods

### Remote sensing data

#### Modis NDVI

We employ the temporal 16-day composite series of MODIS NDVI (MOD13Q1, Collection 6 available on http://earthexplorer.usgs.gov), with a spatial resolution of 250 m. This product is retrieved from atmosphere-corrected^39^, daily bidirectional surface reflectance observations, using a compositing technique based on product quality^40^. We use the 16-day composites to ensure a high probability of a reduced cloud effect on the NDVI pixels retrieved. The entire period from 2001 to 2018 is considered in our analysis of drought. The 16-day data have been averaged to the monthly time step.

#### ASCAT SWI

The ASCAT scatterometer radar is one of the 12 instruments carried by ESA’s METOP-A satellite (launched in 2006), and operates in the C‐band (5.3 GHz), in vertical polarization. Over land surfaces, various approaches have been proposed for estimating surface soil moisture. The Institute of Photogrammetry and Remote Sensing (IPF), Vienna University of Technology (TU‐Wien) has offered a soil moisture product at a spatial resolution of 12.5 km based on ASCAT data since 2007. The product is distributed as a daily dataset by the Copernicus Global Land Service (http://land.copernicus.eu/global/products/swi). The proposed approach is based on a change detection methodology, using normalized multi-incidence radar signals. The derived soil moisture index is between 0 and 1 (0 for dry soils, 1 for very wet surfaces). This product has been validated through analysis in several regions of the globe and by intercomparison with other satellite products. Based on the high repetitivity of these products, the Soil Water Index data (SWI) was derived from m_s_ using the following equation (1). It represents the root-zone soil moisture content in the first meter of the soil in relative units ranging between wilting point and field capacity.

1 $$SWI(t)=\frac{\sum _{i}{m}_{s}({t}_{i}){e}^{-(t-{t}_{i})/T}}{\sum _{i}{e}^{-(t-{t}_{i})/T}}\,\,\,for\,\,\,{t}_{i}\le t$$

where m_s_ is the surface soil moisture estimate from the ASCAT scatterometer at time t_i_. The parameter T, called the characteristic time length, represents the time scale of soil moisture variations in units of time. A T of 20 days has shown the best fit to ground measurements.

The daily data have been averaged to the monthly time step. ASCAT moisture products were validated over the central Tunisia, using continuous ground moisture measurements^15^.

#### Modis LST

In this study, we employ the monthly composite MOD11C3 series, a collection^41^ having a spatial resolution of 0.05°. This product is retrieved from atmosphere-corrected, daily bidirectional surface reflectance observations, using a compositing technique based on product quality. The entire period from 2001 to 2018 was examined for our analysis of drought. We are however aware that LST changes rapidly in space and time owing to the strong heterogeneity of land surface characteristics in vegetation, topography and soil^42^, such that these monthly time-step data might not be the best choice for drought analysis.

### Administrative division

The exact definition of our area is the extent of administrative areas in the three above-mentioned countries where the average normalized difference vegetation index (NDVI) for every month of the last 10 years is above 0.18, hence most of the croplands of those countries falls within this area. Desert regions located in the south of North Africa, with very dry conditions and very limited vegetation covers, are not considered in our study. According to FAOSTAT, 2009, the cultivated areas of Morocco, Algeria and Tunisia cover respectively 9, 8.4 and 5 million hectares. In order to consider spatial behavior, it is more convenient to conduct analyses of the indices’ variations at the administrative region scale. This allows us to retrieve a mean behavior, first to avoid noise due to crop changes at pixel scale, and second, because administrative scale is very useful for the management of drought and the interpretation of results by stakeholders.

The Global Administrative Areas Database (GADM, version 2.8, Nov. 2015) was developed by R. Hijmans and colleagues at UC Berkeley (http://www.gadm.org). For our purpose, we focused on administrative levels, which are relatively homogeneous in size across the three countries of the Maghreb. Thus the levels that were chosen are the provinces for Morocco, the wilayas for Algeria and the governorates for Tunisia. The original data have been unified into a single administrative vector file and simplified for the purposes of display and analysis.

### Drought indices

We use a normalization of the three primitive variables NDVI, SWI and LST for estimation of drought indices and name them VAI, MAI and TAI for Vegetation Anomaly Index, Moisture Anomaly Index and Temperature Anomaly Index.

The VAI^18,43^ is obtained from equation (2).

2 $$VAI=\frac{NDVI-\overline{NDVI}}{{\sigma }_{NDVI}}$$

The temporal standard deviation of NDVI (*σNDVI*) and the NDVI average ($\overline{NDVI}$) are then computed for a temporal period (e.g., one month) using 17 years of satellite data.

The MAI^15^ is obtained from equation (3)

3 $$MAI=\frac{SWI-\overline{SWI}}{{\sigma }_{SWI}}$$

where *σSWI* is the temporal standard deviation of SWI and $\overline{SWI}$ is the SWI average

Finally the TAI is obtained from equation (4) as

4 $$TAI=\frac{LST-\overline{LST}}{{\sigma }_{LST}}$$

where *σLST* is the temporal standard deviation of LST and $\overline{LST}$ is the LST average.

The VAI, MAI and TAI are dimensionless indices ranging theoretically from unlimited negative values (drier than normal) to unlimited positive values (wetter than normal).

The indices are computed for the administrative areas. The most common way of synthesizing raster data to create vector polygons is to rasterize the vector dataset and tag each polygon. The average, sum or other calculations are then computed for the observed value of each polygon. Since the resolution of our input data ranges between 250 meters and 12 km, we preferred to compute a weighted vectorized synthesis. The principle is to vectorize the input raster, combine it with the input administrative vectors and compute the proportion of each pixel belonging to the administrative area. To improve calculation time, the result of crossing vectors and weights are obtained only once, and saved for further calculations.

The mean and standard deviation of satellite products are computed before the final calculation of the anomalies. We conduct further work with a vector of indicators that we have named the drought vector as in equation (5)

5 $$VD=[\begin{array}{c}VAI\\ MAI\\ TAI\end{array}]$$

### Correlation analysis

The correlation analysis can provide valuable information regarding the connection between variables and/or lagged connections. We have used the Pearson standard correlation coefficient, which is a common method for analyzing the intensity of correlation between variables. The correlation coefficient is obtained from equation (6):

6 $$\frac{cov(X,Y)}{{\sigma }_{X}{\sigma }_{Y}}$$

where X is a variable to be tested and Y is another variable possibly lagged in time, *cov* is the covariance and *σ*_*X*_ and *σ*_Y_ are the standard deviations of variables X and Y.

To perform the analysis, we use the data from 2007–2018 where all three variables are available. We then limit the study period to the months of November through May, which cover most of the regional crop development. The Pearson correlation coefficient is then computed using each month of the period for each administrative area.

This is done for the primitive variables NDVI, SWI and LST. We then compute-lagged correlations between NDVI and lagged NDVI, lagged SWI and lagged LST for a lag of one to four months.

### Spatial analysis of drought intensity

Based on the VAI and MAI index estimations over the studied site, we try to retrieve a mapping of drought intensities. The distance to the (0,0) position is computed with equation (7):

7 $$D=\sqrt{VA{I}^{2}+MA{I}^{2}}$$

the circle to which the class belongs is obtained with the threshold S and by limiting the numbers of circles to two, as in equation (8):

8 $$C=\,\min ((\frac{D}{S}),2)$$

each circle is then divided into four quadrants numbered from 0 to 3, which are obtained from the signed angle in radians, as in equation (9):

9 $${\rm{Q}}={\rm{floor}}(({\tan }^{-1}(\frac{VAI}{D},\frac{MAI}{D})/\pi +1)\ast 2)$$

Finally, the class is obtained by combining the circles C and the quadrants Q following equation (10):

10 $$CC=C\ast 4+Q\,with\,CC < 4\,assigned\,to\,class\,1$$

**Figure 9:**
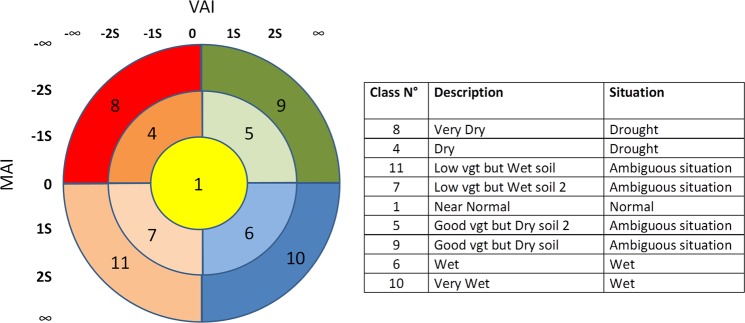
Classification thresholds on the left; class descriptions on the right.

The graphical expression of this distribution is explained in Fig. 9. The proposed classification is fully symmetric. After some empirical tests, we selected a threshold of S = 0.75, which achieved a coherent distribution of situations. This method allows the separation of drought, normal and wet situations, but it also reveals abnormal or ambiguous situations, in particular when the moisture and vegetation anomaly indices are opposed. Situations 7 and 11 should mean that the badly developed vegetation can potentially be invigorated by high soil moisture, while on the other hand situations 5 and 9, with well developed vegetation, could potentially deteriorate owing to abnormally low soil moisture. The classification scheme also allows us to break down the intensity of the situation, for example Very Dry (Class 8) versus Dry (Class 4).

Because the ambiguous classes are very uncommon, Class 9 has been grouped with Class 5 and Class 11 with Class 7. Both are in any case ambiguous situations, in fact Classes 7 and 11 are situations in which the vegetation is underdeveloped but has a wet soil. This situation is most likely to occur when a dry situation is reverting to a normal one. On the other hand, Classes 5 and 9 display well-developed vegetation but dryer soil than normal. If this situation occurs at the beginning of the season, it is possible than a season that was looking promising is reverting to a normal situation, or to a rapid senescence. Note that such ambiguous situations mainly occur in the rainiest portions of the study area, but some cases also involve more semi-arid areas.

### Predictability with analogues

In the technique we employed, the vector used is the drought vector described above. The search area is limited to the studied administrative area itself. For one studied month, a time series of the drought vector VD is constructed for the preceding months. For the months of November and December, the time series are three months long ([sept,oct,nov] and [oct,nov,dec] respectively), while the other months are tested for the [nov-month_to_test] time series. For example, for the month of March, the time series are [nov,dec,jan,feb,mar]. The 2-dimensional VD is flattened to one dimension on the time dimension as in equation (11), which is possible because of the earlier normalization.

11 $$V{D}_{i}=[\begin{array}{c}VA{I}_{i}\\ MA{I}_{i}\\ TA{I}_{i}\end{array}]\,= > \begin{array}{ccc}V{D^{\prime} }_{i}=[VA{I}_{i} & MA{I}_{i} & TA{I}_{i}]\end{array}$$

VD’ is compared to other years’ VD” using the nearest neighbor method with Euclidean distance (*VD′, VD*”) ”s in equation (12), where *i* are the items of the flattened drought vector (VAI, MAI and TAI) for the tested period:

12 $$d(VD\text{'},VD^{\prime\prime} )=\sqrt{{\sum }_{i=1}^{n}{(V{D^{\prime} }_{i}-V{D}_{i}^{\prime\prime} )}^{2}}$$

This approach allows us to compute a score and to rank the various years. In order to analyze the value of this approach for predictability, we compute the objective function as the first year found as the analogue of April, and then compare the rankings for the months of November through April, which means that 1530 tests are carried out (17 years × 90 areas) for each of the 6 months tested.

## Data Availability

All the data used in this article are freely available online from http://www.gadm.org, http://earthexplorer.usgs.gov, http://land.copernicus.eu, http://chg.geog.ucsb.edu/data/chirps.
